# Corticosteroid therapy for the management of paradoxical inflammatory reaction in patients with pulmonary tuberculosis

**DOI:** 10.1007/s15010-020-01430-7

**Published:** 2020-04-24

**Authors:** Macky M. Done, Onno W. Akkerman, Wud Al-Kailany, Wiel C. M. de Lange, Gonda de Jonge, Johanneke Kleinnijenhuis, Riejanne Stienstra, Tjip S. van der Werf

**Affiliations:** 1 Department of Pulmonary Diseases and Tuberculosis, University of Groningen, University Medical Center Groningen, Groningen, The Netherlands; 2 Department of Medical Imaging, University of Groningen, University Medical Center Groningen, Groningen, The Netherlands; 3 Department of Internal Medicine, Division of Infectious Diseases, University of Groningen, University Medical Center Groningen, Groningen, The Netherlands

**Keywords:** Tuberculosis, Paradoxical reaction, Corticosteroids, SIRS

## Abstract

**Background:**

Paradoxical reaction after the initiation of tuberculosis treatment is defined as increased inflammation following effective antimycobacterial treatment. This is a phenomenon that can severely complicate a patient’s recovery, potentially leading to further morbidity and residual deficits. Paradoxical reaction remains poorly understood regarding its pathophysiology and management. Only a limited number of reports look critically at the available therapeutic options, with evidence of the efficacy of prednisolone therapy being primarily limited to extrapulmonary PR only.

**Case:**

We describe two HIV negative patients who were admitted to our department with pulmonary tuberculosis, presenting with inflammatory patterns attributable to PR and their response to adjunctive steroid therapy.

**Discussion and Conclusions:**

The presented cases further highlight the need for immunological studies and randomized trials for corticosteroid therapy are needed to better understand this phenomenon as well as provide an evidence-base for anti-inflammatory treatment. Furthermore, by means of this case series, we are also able to highlight the potential variability in the symptomatology of the lesser known PR phenomenon, in which we observed a hypotensive shock-like syndrome not previously described in literature.

## Introduction

*Mycobacterium tuberculosis (Mtb)* continues to be a daunting threat despite highly effective and standardized antimicrobial treatment [1]. Although harmless in its dormant form, *Mtb* is pleiotropic, and with active replication, tuberculosis (TB) can be lethal [2]. Indeed, worldwide it is currently recognized as the number one cause of death due to infectious disease with 1.5 million people dying out of the estimated 10 million with active disease in 2018 alone [3, 4]. This stems largely from the virulence of this pathogen, but also the host response may be detrimental in the form of a paradoxical reaction (PR). This phenomenon constitutes the occurrence of clinical and/or radiographic worsening despite effective reduction of bacterial load [5–7]. This diagnosis should be considered from 2 weeks after anti-microbial therapy initiation, but becomes more likely after 2 months [6]. It is a diagnosis of exclusion—failure of therapy, super infection and drug-mediated toxicity must first be considered [6]. We describe two HIV-negative patients that were treated for pulmonary TB who later presented with PR. We report their response to adjuvant corticosteroid therapy via clinical observation and radiographic imaging.

## Case reports

### Case A

**Fig. 1:**
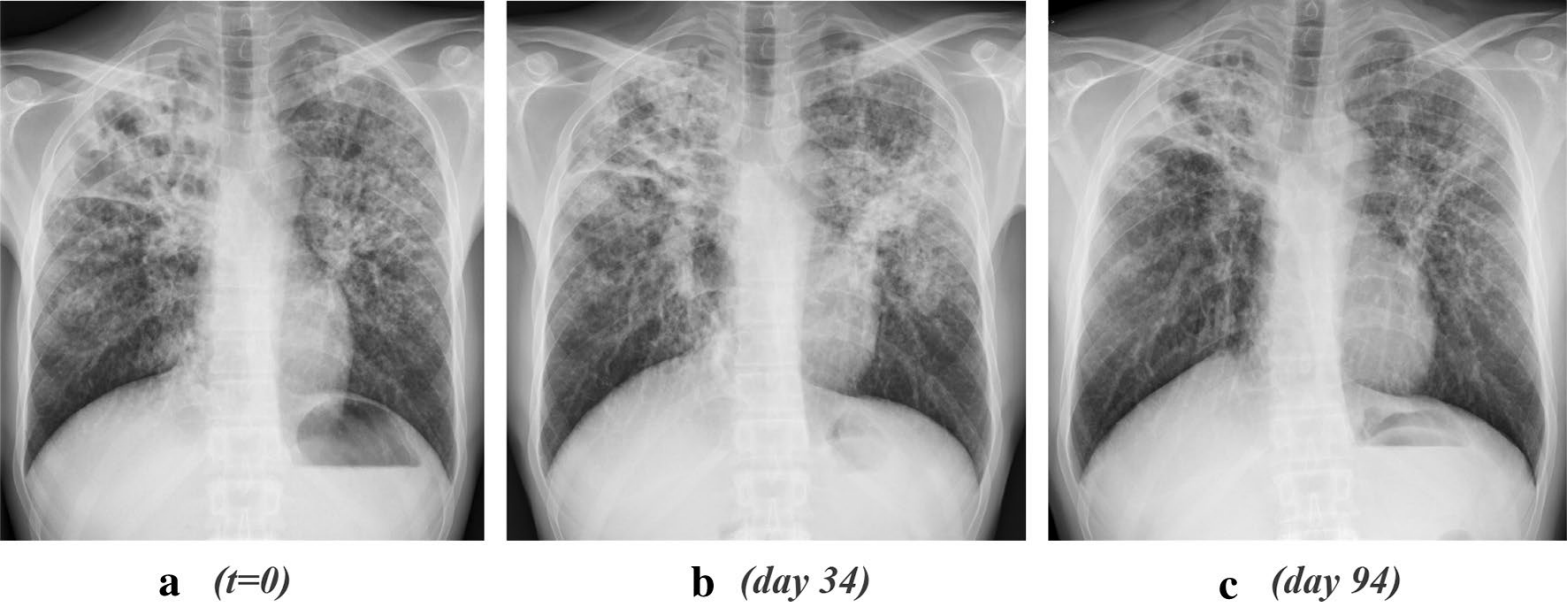
**a**–**c** Cavitary changes RUL and bronchogenic spread to RML and LUL; **b** 1 month later, a transient increase in infiltrative changes are evident in the LUL and RML that have subsided at month 3 (**c**) and month 4 (not shown). *RUL* right upper lobe, *LUL* Left upper lobe, *RML* right medial lobe

Patient A is a 34-year-old male of Moroccan origin who was homeless and known with substance abuse. He had no recorded previous medical history. He presented with the following symptoms: a cough for 7 weeks before admission (without hemoptysis), loss of 18.2 kg body weight within a 3-month period and night sweats matched with severe depletion of muscle mass and fat reserves. On physical examination, fine crackles were present over the lungs. There were no symptoms to suggest extrapulmonary involvement. Body temperature was 38.1 ℃. The heart rate upon admission was 110 bpm with a blood pressure 105/75 mmHg. His BMI was 17.2 kg/m^2^. Upon admission, chest X-rays (see Fig. 1 below) showed infiltrates in both lungs. He was anemic (Hb 6.2 mmol/L) with MCV 75.4 fL; C-reactive protein (CRP) was elevated 73 mg/L; leukocyte count 11 × 10^9^/L and 14.1 × 10^9^/L the day after admission. This patient was not known to be diabetic and blood glucose values matched this conclusion. Fluorescent staining of sputum smear microscopy showed abundant acid-fast bacilli, and sputum culture later revealed *M. tuberculosis*. Polymerase chain reaction (PCR) targeting IS 6110 was positive, consistent with *Mtb*. He tested negative for HIV as well as hepatitis B and C co-infections. Bronchoscopic mediastinal lymph node and lung tissue cytology specimens displayed the presence of polynuclear giant cells in the mediastinal lymph nodes as well as the lungs, suggestive of granulomatous necrotizing inflammation.

The patient was started on isoniazid, rifampicin, ethambutol, and pyrazinamide (HRZE). 10 days later, Isoniazid was replaced by moxifloxacin after the results of drug susceptibility testing (DST) revealed isoniazid mono-resistance. A month after the initiation of therapy, he developed pyrexia and night sweats associated with a failure to gain weight after initial clinical improvement. Culture negativity was achieved after 2 months of therapy after a progressive reduction in the bacterial load present in the sputum. Blood and urine cultures were negative. The symptoms and signs of increased inflammation including deterioration of the chest radiograph were highly suggestive of paradoxical inflammation. He was started on a daily dose of 30 mg of prednisolone while TB treatment was continued. His clinical response was favorable; his temperature settled, his dyspnea resolved, and after 2 weeks, steroids could be tapered, first to 20 mg/day when his symptoms started to subside, and subsequently the dosage was further reduced and stopped after a total of 37 days. His clinical course was ultimately uneventful.

### Case B

**Fig. 2:**
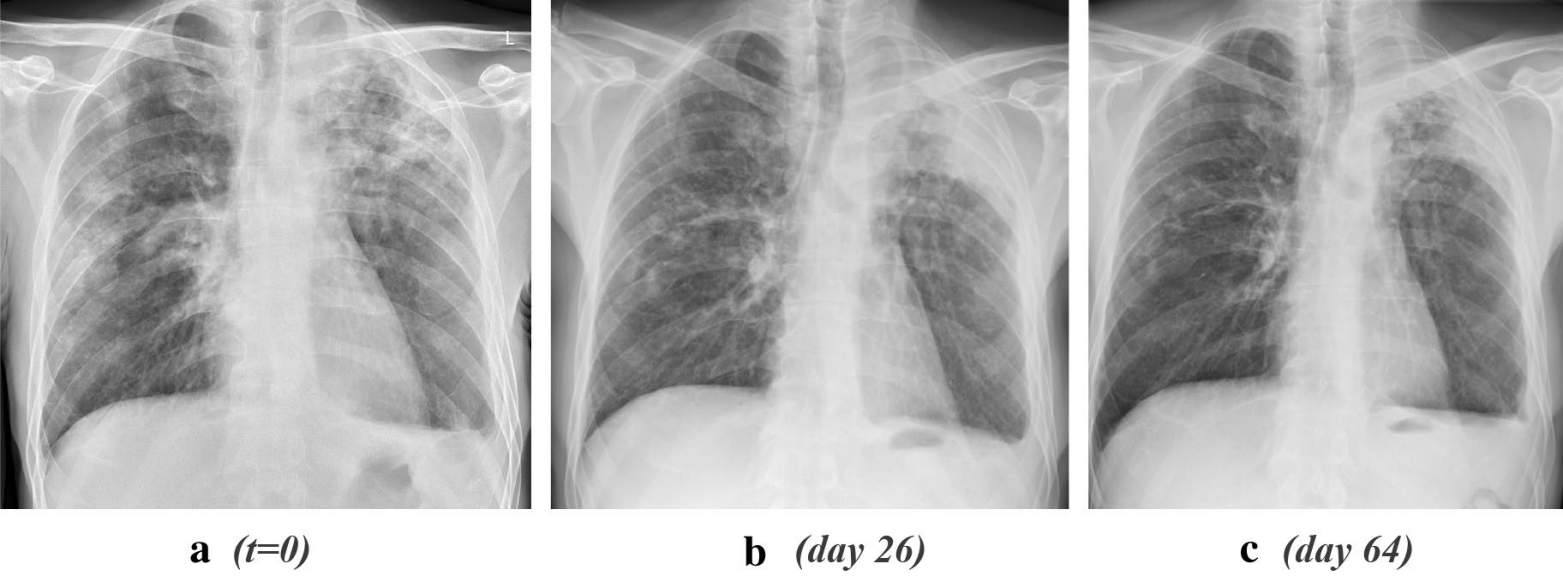
** a**–**c** Chest X-ray comparison of patient B on day 1(left), day 26 (middle) and day 64 (right). This sequence of radiographs shows an increase of infiltrative consolidation and pleural thickening in the LUL, corresponding to the manifestation of PR symptoms and a subsequent reversal of these changes (**c**) following corticosteroid therapy initiation

**Fig. 3:**
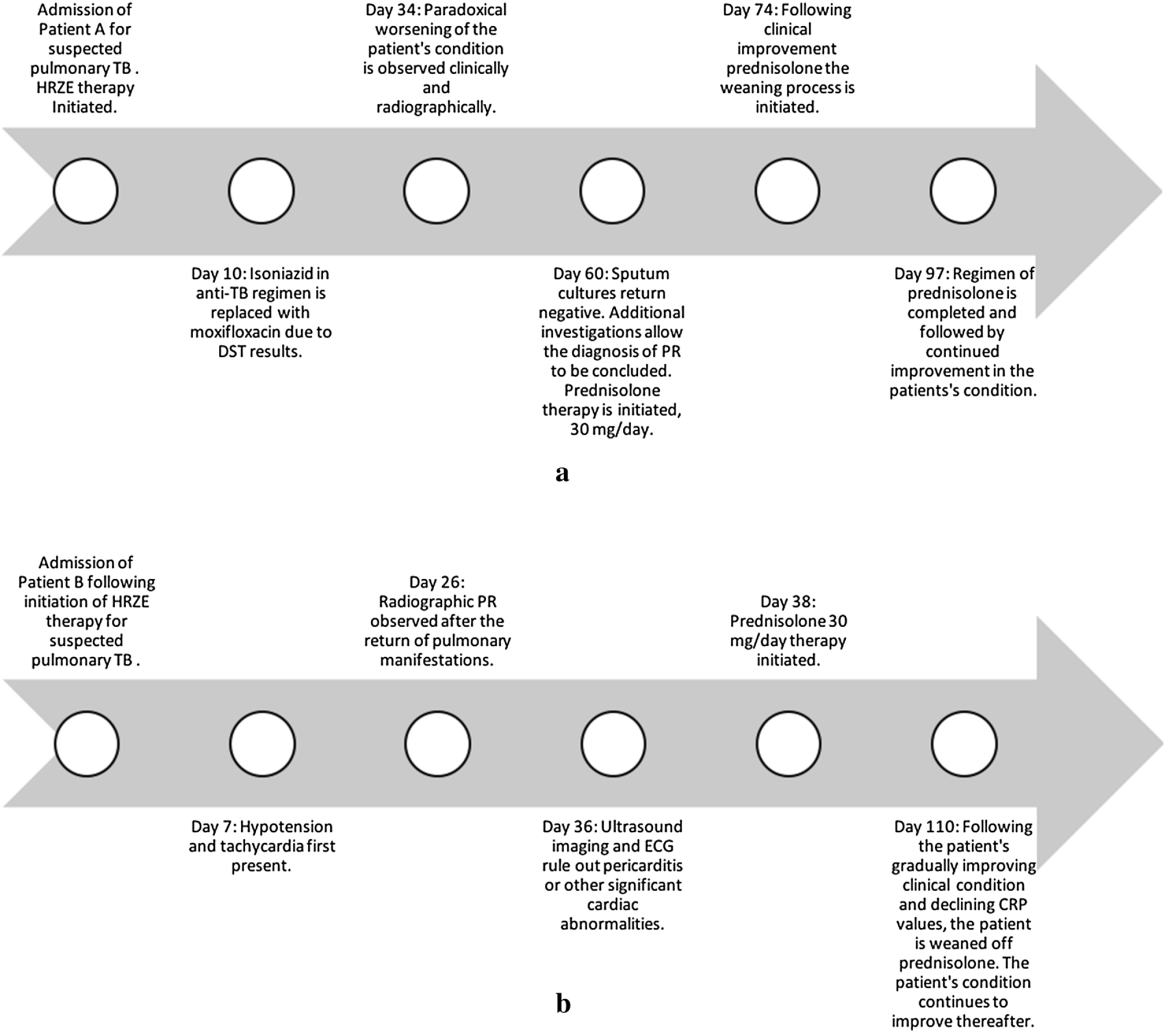
**a** Timeline of patient A. **b** Timeline of patient B

Patient B is a 48-year old man originating from Poland with a previous history of chronic alcohol abuse. He presented in another health facility with a stabbing chest pain, dyspnea, chronic cough (with the presence of hemoptysis) and fever. In the month prior, he had observed a weight loss of 10 kg with significant muscle wastage impacting his ability to walk unaided. Upon auscultation, vesicular breath sounds were heard, without crepitations. His temperature was 40.1 ℃, blood pressure 117/78 mmHg, heart rate 93 bpm and respiratory rate 24 cycles/min. He was anemic with Hb 6.5 mmol/L, a leukocyte count of 6.5 × 10^9^/L and an elevated CRP of 195 mg/L. His blood glucose was 5.1 mmol/L at presentation and remained in the normal range during admission. Ziehl–Neelsen stained sputum microscopy showed abundant acid-fast bacilli. He was started on HRZE combination therapy; sputum cultures later showed fully susceptible *Mtb*. Vitamin supplementation included pyridoxine and thiamine, targeting the patient’s alcohol-related nutritional deficiencies. When transferred to our facility, further radiographic imaging displayed extensive pulmonary infiltrates bilaterally. Initially, he vomited and had diarrhea and we suspected central nervous system—or perhaps intestinal or peritoneal involvement of tuberculosis. A brain MRI displayed no abnormalities allowing for the exclusion of brain involvement. A week later, he deteriorated further with a change in his vital parameters, including hypotension (80/50 mmHg), and tachycardia (145 bpm). Nevertheless, a gradual reduction in his dyspnoea and CRP was observed during the first weeks despite the hypotension and tachycardia persisting. However, a reversal of these clinical improvements was seen in the same time frame that followed. We suspected an intercurrent nosocomial infection with sepsis, and blood, sputum and urine cultures were taken but remained negative. Concurrent sputum mycobacterial cultures showed a gradual reduction in the mycobacterial load. Furthermore, with each sputum culture, an increased time to positivity was observed until finally becoming culture negative after 58 days of the anti-TB therapy. An ECG showed sinus tachycardia without any other abnormality. Cardiac ultrasound did not show pericardial effusion or other abnormalities. Exercise testing showed poor exercise tolerance (50 W), but no desaturation was noted. The chest X-ray (see Fig. 2) had worsened and with CRP increased to 276 mg/L and leukocyte count of 10.4 × 10^9^/L, a severe PR was suspected, and he was now started on 30 mg prednisolone per day. The response to steroids was positive, but more attenuated and subtle compared to patient A. The added corticosteroid could eventually be tapered over a 3 month period (Fig. 3), in which he showed gradual improvement, with a mild tachycardia (approximately 100 bpm) persisting for a month following prednisolone’s cessation before stabilizing. His sputum cultures remained negative and his body weight increased, while CRP gradually declined to below 20 mg/L. Upon continued improvement, displayed by subsequent imaging studies and his clinical condition, the patient was discharged from the department. However, given our patient’s unsatisfactory rate of progress throughout his admission, an extended 9 month anti-tuberculosis therapy regimen was implemented, the remainder of which was continued on an outpatient basis using DOT.

## Discussion

In both patients, we diagnosed PR with a worsening of the clinical condition and radiographic pathology. Corticosteroids worked well for patient A, but for patient B this treatment was not necessarily overwhelmingly effective to control all signs and symptoms of PR such as the cardiovascular dysfunction which persisted. In the past, corticosteroids have been in use to manage a variety of forms of extrapulmonary TB [8, 9]. Persisting hypotension suggested cardiac (or pericardial) involvement [10]; or overwhelming TB with sepsis [11, 12]. Although *Mtb* itself is effectively killed by appropriate antimicrobial therapy, an exaggerated immune response elicited by the release of virulence factors from the cell wall—possibly mediated via IL-1 and TNF pathways—may be explanatory for the development of PR [11–13]. This mechanism is also able to clarify the therapeutic effect exhibited by TNF-a inhibition, in patients who do not respond to corticosteroids [14, 15]. Currently, much of the evidence available evaluating the efficacy of corticosteroids in treating PR assesses this within the context of TB IRIS (immune reconstitution inflammatory syndrome). In this group, the preventative value of early administration of prednisone in the development of PR has recently been demonstrated [16]. This intervention is yet to be applied to those that do not belong to this risk group of patients with IRIS following antiretroviral therapy.

For patients such as those we have described, formal trials are required to shed light on the possible benefits and harms caused by steroids and anti-TNF immunosuppressive therapies as well as other alternative approaches. The benefits of all of these interventions have been suggested in case reports without evidence based on comparative studies or randomized trials [17]. Meanwhile, mechanistic immunological studies could help clarify the underlying pathways involved. Currently, we study the incidence of PR in TB, and explore anti-inflammatory intervention with a macrolide aiming to dampen excessive immune activation and structural damage during TB therapy [NTC03160638]. In summary, we describe excessive inflammatory response following initiation of effective antimicrobial therapy for TB. We argue that improved understanding and formal studies are needed to provide evidence for the treatment of PR.
